# Caregiver Feeding Behaviours and Child Dietary Diversity and Growth in Rural Bangladesh

**DOI:** 10.1111/mcn.13781

**Published:** 2024-12-13

**Authors:** Zaynah T. Chowdhury, Kristen M. Hurley, Saijuddin Shaikh, Sucheta Mehra, Hasmot Ali, Abu Ahmed Shamim, Parul Christian

**Affiliations:** ^1^ Department of International Health, Center for Human Nutrition Johns Hopkins Bloomberg School of Public Health Baltimore Maryland USA; ^2^ JiVitA Project Gaibandha Bangladesh

**Keywords:** Bangladesh, child growth, dietary diversity, feeding behaviours, responsive feeding, South Asia, undernutrition

## Abstract

This study examined relations between caregiver feeding behaviours, child dietary diversity and anthropometry at 24 months of age in rural Bangladesh. Twenty‐four hours dietary recall, weight and length data were collected on 4733 children. Factor analysis was applied to an 11‐item caregiver feeding behaviours scale administered at 24 months, revealing two constructs: responsive/involved (five items) and forceful (six items); each dichotomised to reflect low and high use. Stunting (length‐for‐age *Z*‐score < −2), wasting (weight‐for‐length *Z*‐score < −2) and underweight (weight‐for‐age *Z*‐score < −2) were defined using international growth reference standards. Associations between feeding behaviours, dietary diversity score (DDS, food groups consumed; range 0–7) and anthropometric indicators were examined using multivariable linear or logistic regression models, adjusting for study design, confounders and intervention arm. Mean (SD) age of children in the study was 24.1 (0.3) months. Mean (SD) DDS was 3.7 (1.4), with 55% of children meeting minimum dietary diversity (MDD, DDS ≥ 4). Stunting, wasting and underweight were 40%, 19% and 42%, respectively. Use of high responsive/involved feeding behaviours (reported in 71% of mothers) was associated with higher DDS (0.09, 95% confidence interval [CI]: 0.001, 0.17) and higher odds of achieving MDD (OR: 1.17; 95% CI: 1.02, 1.33) but not with anthropometric outcomes. Use of high forceful feeding behaviours (reported in 34% of mothers) was associated with lower DDS (−0.12, 95% CI: −0.21 to 0.04), lower odds of achieving MDD (OR: 0.82, 95% CI: 0.72, 0.93), and higher odds of underweight (OR: 1.38, 95% CI: 1.22, 1.56) and wasting (OR: 1.55, 95% CI: 1.33, 1.81). In Bangladesh, responsive/involved feeding was associated with higher child dietary diversity whereas forceful feeding was associated with lower dietary diversity and undernutrition. Future research is needed to understand causality and test the effect of responsive feeding interventions on the promotion of child growth.

## Summary

- Although access to foods and nutrients plays a central role in early nutrition, variability in feeding behaviours also influence children's eating patterns.
- Findings suggest that in the context of high undernutrition in rural Bangladesh, responsive/improved feeding behaviours were associated with improved dietary diversity at 2 years, while nonresponsive feeding behaviours (especially forceful) were associated with poor growth and dietary diversity among young children.
- Further research is needed to understand the directionality and test the effect of responsive feeding interventions on the promotion of healthy dietary patterns and growth in young children.

## Graphical abstract

In the context of high undernutrition in rural Bangladesh, responsive/improved feeding behaviours were found to be associated with improved dietary diversity at 2 years, while nonresponsive feeding behaviours (especially forceful) were associated with poor growth and dietary diversity among young children. Suaahara I. (2011). Responsive Feeding ‐ Encouraging a child to eat ‐ 01 ‐ Nepal. Accessed from the USAID Advancing Nutrition‐UNICEF IYCF Digital Image Bank (iycf.advancingnutrition.org).

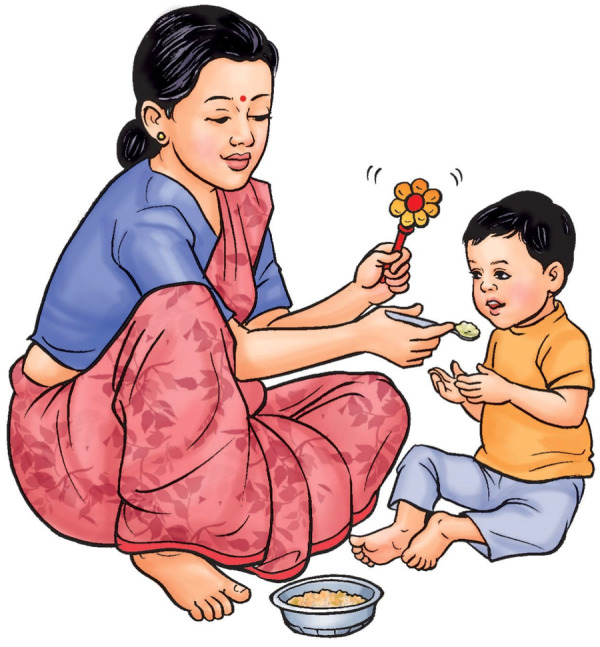

## 1 Introduction

Global estimates of child growth measures reveal a concerning number of children at risk for or privy to inadequate diets and/or exposure to infection, both of which can exacerbate undernutrition (Victora et al. 2021). The burden of stunting (height‐for‐age) and wasting (weight‐for‐height < −2 *z*‐scores below the reference median) remains higher in low‐income countries, where they persist as public health issues (Victora et al. 2021). Both are associated with an increased risk of childhood morbidity and mortality and can impact health and productivity later in life. For stunted children, these detriments are characterised by cognitive deficits, metabolic dysfunction, poorer performance in school and work, and adverse pregnancy outcomes that perpetuate the cycle of stunting into future generations (Dewey and Begum 2011; Victora et al. 2021). More than half the global number of stunted and wasted children live in Asia; in Bangladesh, 21.9% of children less than 5 years of age are underweight (National Institute of Population Research and Training NIPORT and ICF 2020; United Nations Children's Fund UNICEF 2021). In rural areas of Bangladesh, 32.8% of children are stunted, reflecting an even higher burden among rural populations and of the lowest socioeconomic status (National Institute of Population Research and Training NIPORT and ICF 2020).

Exclusive breastfeeding is recommended in the first 6 months of life after which children require appropriate quantity and quality of complementary foods alongside breast milk for optimal growth and development (Dewey and Begum 2011). During feeding, infants and young children need appropriate encouragement and need to be presented with a variety of foods to ensure they consume sufficient amounts of foods with different flavours, texture and consistency, which vary by age as taste and flavours are learnt and by breastfeeding status (Pérez‐Escamilla, Jimenez, and Dewey 2021). In addition, even when food availability is ensured, caregivers may lack knowledge regarding feeding young children, or cultural practices and inappropriate advice may drive inappropriate infant and young child feeding practices (Pelto, Levitt, and Thairu 2003). In the context of low‐middle income settings, lack of diversity, inappropriate consistency (e.g., traditional thick gruels), low nutrient density and insufficient quantity of foods are major contributing factors to inadequate diets and intake among infants and young children during the complementary feeding period (Dewey and Begum 2011). Underlying these is a lack of access to nutritious foods (Affleck and Pelto 2012), insufficient counseling and knowledge of optimal feeding practices among caregivers, as well as the lack of a supportive social environment (Lutter et al. 2011). Lack of dietary diversity is a particularly severe problem in children living in impoverished populations where diets are predominantly starch‐based and include few or no animal sources or fruits and vegetables (Arimond and Ruel 2004). These diets often result in macro‐ and micronutrient deficiencies that take a significant toll on the health and development of children, particularly those under 5 years of age (Black et al. 2013).

Another aspect that lacks exploration is the relationship between feeding behaviours and lack of diversity in diets of children living in LMICs. Caregiver–child interactions or feeding behaviours refer to the interactive behaviours between caregivers and children that occur during meals (Black and Aboud 2011). Responsive feeding is characterised by a give and take relationship between caregiver and child, where the caregiver is feeding the child based on the child's cues of hunger and satiety (WHO and UNICEF 2003). Nonresponsive feeding behaviours are characterised by a lack of developmentally appropriate/sensitive reciprocity between the caregiver and child, through excessive caregiver control (forcing/pressuring or restricting food intake), excessive parental passivity or child control (indulgence) or caregiver disregard (uninvolved) (Black and Hurley 2010). Responsive feeding is also included as a recommendation in the guide for complementary feeding of the breastfed child (Dewey 2003). Until recently, undernutrition in young children has focused largely on foods and nutrients with attention on the quality, diversity and amount of food being offered to children (Affleck and Pelto 2012). Increasingly, however, there is attention being paid to the role of caregiver feeding behaviours (Affleck and Pelto 2012), particularly in low‐ and middle‐income settings (Bentley, Wasser and Creed‐Kanashiro 2011; Frith et al. 2009).

While responsive feeding messages are commonly included in nutrition and/or complementary feeding interventions (Bhandari et al. 2004; Penny et al. 2005; Schroeder et al. 2002), limited studies have looked at the associations between caregiver feeding behaviours (i.e., responsive or nonresponsive feeding) and child dietary diversity and undernutrition. In a rural, Bangladeshi context, where undernutrition is high, we designed a study to address these questions. The aim was to design and implement a structured, interview‐based instrument to capture caregiver feeding behaviours in an on‐going complementary food supplementation trial (Christian et al. 2015) in which dietary intake and anthropometry were assessed at 24 months of age. We report here the relationship between caregiver feeding behaviours captured using this instrument and dietary diversity and nutritional status among 24‐month‐old children.

## 2 Methods

### 2.1 Setting and Study Population

This study was nested within a cluster‐randomised controlled supplementation trial, which compared the efficacy of four complementary food supplements and their impact on growth, length‐for‐age *z*‐score (LAZ), weight‐for‐length *z*‐score (WLZ) and morbidity, among children from 6 to 18 months of age, in addition to provision of nutrition counseling to all participants versus a control group, which received nutrition counseling alone (Christian et al. 2015). A 24‐month follow‐up was planned to examine the sustained impact of the intervention after feeding had been discontinued at 18 months of age.

The trial was conducted in a study area comprising 19 unions (lower‐most administrative unit of the local government system) within the rural areas of two northwestern districts of Bangladesh, divided into 596 community clusters. The study area spans approximately 435 km^2^, with a population of about 650,000 people (Labrique et al. 2011). Most of the population is Muslim, with a poor, agrarian economy (Christian et al. 2015; Labrique et al. 2011). The data in this study were collected as part of the parent trial (Christian et al. 2015).

### 2.2 Data Collection

All study participants were provided home‐based, age‐specific nutrition counseling focusing on infant and young child feeding, health and hygiene provided by trained counsellors using the Alive and Thrive (www.aliveandthrive.org) modules (Christian et al. 2015).

### 2.3 Socioeconomic Indicators

Data on socioeconomic variables including parental age, occupation, education, household asset ownership (cattle, electricity connectivity, land, type of latrine used), household food insecurity, household size, child sex and child age were collected by trained project interviewers at the time of enrollment at 6 months of age. Caregiver perceptions of household food insecurity were assessed using a nine‐item Food Access Survey Tool (FAST) (Na, Gross, and West 2015).

### 2.4 Dietary Diversity

**Figure 1:**
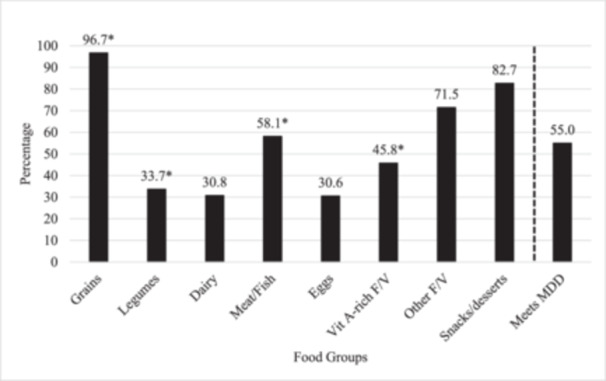
Consumption of food groups and proportion who meet MDD using 24‐h dietary recall data in children at 24 months of age (*n* = 4733). *Note:* Bars indicate the percentage of children consuming each food group at 24 months, as collected by the 24‐h dietary recall. The last bar indicates the percentage of children meeting MDD (DDS ≥ 4). The snacks/desserts group was not included in the DDS. *Indicates groups in which intake differed significantly (*p* < 0.05) by feeding behaviour. After adjustment for confounding variables, responsive feeding was associated with a higher consumption of meat (adjusted OR: 1.19, 95% CI: 1.04, 1.36). Conversely, forceful feeding was associated with lower consumption of grains, legumes and vitamin A‐rich fruits/vegetables (adjusted OR: 0.70, 95% CI: 0.50, 0.98; adjusted OR: 0.86, 95% CI: 0.75, 0.99; adjusted OR: 0.71, 95% CI: 0.63, 0.81, respectively). DDS, dietary diversity score; F/V, fruits and vegetables; MDD, minimum dietary diversity; OR, odds ratio; Vit A, vitamin A.

Children's dietary diversity was assessed by creating a seven‐group Dietary Diversity Score (DDS) according to UNICEF guidelines at the time of the study (WHO, USAID, UNICEF, IFPRI, FANTA‐2, and AED 2010). Diet data were collected using a semi‐structured 24‐h dietary recall administered during the 24‐month follow‐up interview. All foods reported on the 24‐h dietary recall were categorised into the following food groups: (1) grains, (2) legumes, (3) dairy, (4) meat, (5) eggs, (6) vitamin‐A rich fruits and vegetables or (7) other fruits and vegetables. If the child consumed more than one food item that fell within a certain group (e.g., rice and bread), they were only given one point for that group. If the child consumed a food that fell into multiple groups, (e.g., khichuri, a rice and lentil porridge), they received a point for each group. The DDS was constructed by summing the total number of groups that were reportedly consumed by that child during the 24 h before the interview. DDS was treated as a continuous variable and was also dichotomised to create a variable for Minimum Dietary Diversity (MDD), defined as DDS ≥ 4 (WHO, USAID, UNICEF, IFPRI, FANTA‐2, and AED 2010). An 8th group comprising snacks/desserts was created and examined separately from the DDS and MDD and presented descriptively in this study. Each of the eight food groups was treated as a dichotomous variable, defined as either ‘ate’ or ‘did not eat’. Individual food group consumption was examined in relation to each exposure (Figure 1). Details on the dietary assessments have been published (Chowdhury et al. 2020).

### 2.5 Growth

Weight was measured using a Tanita baby weighing scale with 10 g precision (model BD585, Tanita Corporation of America, Arlington Heights, IL, USA) and length was measured with 0.1 cm precision using a locally manufactured length board standardised against the Shorrboard (Weight and Measure LLC, Olney, MD, USA) at the 24‐month home visit. Weights and lengths were converted to *z*‐scores using the WHO child growth standards (WHO Multicentre Growth Reference Study Group and de Onis 2006).

### 2.6 Feeding Behaviours and Scale Development

We developed the Caregiver Feeding Behaviour Questionnaire (CFBQ), an 11‐item interview‐based caregiver‐reported questionnaire to assess feeding behaviours. The questionnaire was adapted from the Child Feeding Questionnaire (Birch et al. 2001), the Infant Feeding Styles Questionnaire (Thompson et al. 2009) and the Toddler Feeding Behaviours Questionnaire (Hurley et al. 2013), each based on theories of parenting and feeding. Each question elicited responses on a five‐point Likert scale as follows: 0 (never), 1 (seldom), 2 (half the time), 3 (most of the time) and 4 (always). The initial questionnaire comprised 53 items created to measure the five dimensions of parenting with respect to feeding behaviours (responsive, forceful, restrictive, indulgent and uninvolved). All items were translated into the local language, Bangla, and then reviewed with local female study interviewers who are familiar with the context. Two‐items thought to lack relevance or clarity for this context were removed.

The CFBQ was piloted in a sample of mothers (*n* = 139) of children aged 15–24 months. Based on respondent feedback and an initial exploratory factor analysis (EFA), 38 items were dropped due to low between‐subject variance, low relevance or clarity reported by pilot sample, and low factor (> 0.30) or double loadings. The final questionnaire administered to the study population (*n* = 4733) had 13 items.

### 2.7 Factor Analysis

EFA was conducted to identify feeding factors. We used an orthogonal rotation and eigenvalues > 1 (Netemeyer, Bearden, and Sharma 2003). The number of factors was informed by examination of a scree plot. Single‐item deletions (*n* = 2) were based on predefined, systematic procedure defined by factor loadings (> 0.30), double loadings, low variance and theory (Netemeyer, Bearden, and Sharma 2003).

EFA revealed three empirically derived constructs of feeding in this region: responsive/involved (five items), forceful (six items) and indulgent (two items). Due to only two items loading with the indulgent factor, a final set of 11 items was used to produce two factors: responsive/involved (range: 3–20; median: 16) and forceful (range: 0–24; median: 7) (Table 1). The Cronbach's *α* coefficient of the responsive/involved factor and forceful factor were 0.44 and 0.80, respectively. The EFA was run again dropping two responsive/involved low‐loading items one at a time and then together, which resulted in a responsive/involved factor Cronbach's *α* of 0.44, 0.52 and 0.59, respectively. Based on no or relatively low item‐deletion improvements in the Cronbach's *α*, all five items were retained due to theoretical reasoning. In addition, the split‐half reliability coefficient (Spearman–Brown coefficient) of the entire 11‐item scale (responsive/involved plus forceful) was good, with a coefficient of 0.80, further justifying the inclusion of all responsive/involved feeding items.

**Table 1:** Factor‐loading matrix of identified feeding behaviours.

Description of individual feeding behaviour items as grouped by factor	Factor loading
**Factor 1. Responsive/involved feeding (*n*** = **5)**	
How often do you know what your toddler eats throughout the day?	0.63
How often do you know when your toddler eats throughout the day?	0.61
How often can you tell when your toddler is full?	0.40
How often can you tell when your toddler does not like the food?	0.28
How often do you eat with your toddler?	0.15
**Factor 2. Forceful feeding (*n*** = **6)**	
How often do you force your toddler to eat?	0.78
How often do you beg or plead with your toddler to eat?	0.69
How often do you physically struggle with your toddler to get him/her to eat?	0.67
How often do you yell at or threaten your toddler to be sure he/she eats enough?	0.63
How often do you talk to your toddler during meals?	0.61
How often does your toddler walk around while eating or drinking?	0.39

### 2.8 Statistical Analyses

Descriptive statistics were used to explore the distribution, frequencies, means and SD for study variables. SES indicators were used to create a living standards index (LSI) (Gunnsteinsson et al. 2010) that was categorised into quartiles. Using the responses from the FAST (Na, Gross, and West 2015) tool, household food insecurity (HFI) was categorised into three groups to represent food secure (HFI = 9), food insecure (HFI = 10–15) or severely food insecure (HFI ≥ 16). Stunting, wasting and underweight variables were created by dichotomizing LAZ, WLZ and WAZ at a cutoff of −2 *z*‐scores relative to their respective reference medians. Responsive and forceful feeding variables were both dichotomised to reflect low and high feeding behaviours, with approximately two‐thirds of the sample reporting optimal feeding behaviours for both constructs (high responsive [70%], low forceful [66%]) (Table 2). The responsive/involved factor will be referred to as ‘responsive’ in the rest of the manuscript.

**Table 2:** Matrix of response frequency for feeding behaviour responses (*n* = 4733).

	High responsive, *n* (%)	Low responsive, *n* (%)
High forceful	1123 (23.7)	485 (10.3)
Low forceful	2215 (46.8)	910 (19.2)

Univariate regression analyses were conducted to explore convergent validity and determine potential confounding relationships between feeding behaviours and demographics. Regression models were run with high responsive/low forceful as the reference category. For the continuous outcomes of DDS, WAZ, LAZ and WLZ, we used a linear regression model to estimate the differences between caregivers classified as using high versus low force adjusted for confounding factors. For dichotomous outcomes of MDD, consumption of individual food groups, stunting, wasting and underweight, logistic regression analyses were conducted to estimate odds ratios (OR) and 95% confidence intervals (CI), also adjusted for confounders. HFI was dichotomised and examined as an effect modifier between feeding behaviour and WLZ and WAZ.

All analyses were adjusted for potential confounders and intervention arm, and a variance estimator was included to account for clustering at the sector level given the possibility that respondents living in the same place could be responding to the CFBQ in more similar ways. Statistical significance was set at *p*‐value < 0.05. Statistical analyses were conducted using Stata 14.0 (StataCorp, College Station, TX, USA).

### 2.9 Ethical Approval

All data collection instruments and procedures were approved by the Ethical Review Committee at icddr,b, Bangladesh and the Institutional Review Board of the Johns Hopkins Bloomberg School of Public Health, MD, USA. Written parental consent was obtained at the time of enrolment; all participants were informed that their participation is voluntary and were assured of the confidentiality of the data collected.

## 3 Results

Out of 5411 children in the original trial, 4972 (92%) completed the 24‐month follow‐up interview out of which 4733 mother–child dyads were included in the final analysis after excluding 239 children with missing feeding behaviour data.

### 3.1 Characteristics of Study Participants

Parental, child and household characteristics are described in Table 3. About a quarter of households were severely food insecure (28%), half owned cattle (53%), three‐fourths owned land (71%), about a third had electricity (31%) and 83% had access to an improved sanitation facility. About three‐quarters of mothers had some education (76%), but only 11% were educated beyond high school. Most mothers had no occupation (88%), and the ones who did were service workers (11%).

**Table 3:** Baseline parental, child and household characteristics of enrolled children at 24 months (*n* = 4733).

	Overall
Parental and household characteristics	Mean (SD)
Household size	5 (1.9)
Paternal age (years)	31.1 (7.2)
Maternal age (years)	24.2 (5.4)
Child age (months)	24.1 (0.3)
LSI	0.009 (1.0)

	*n* (%)
Child sex, male	2424 (50)
Maternal education	
No education	1140 (23.6)
Class 1–9	3163 (65.4)
SSC passed	238 (4.9)
11 years and above	294 (6.1)
Paternal education	
No education	1732 (36.8)
Class 1–9	2236 (47.5)
SSC passed	254 (5.4)
11 years and above	489 (10.4)
Maternal occupation	
No occupation	4272 (88.4)
Field labourer	32 (0.7)
Service worker	520 (10.8)
Other	10 (0.2)
Paternal occupation	
No occupation	52 (1.1)
Field labourer	2331 (48.8)
Service worker	2368 (49.6)
Other	27 (0.6)
HFI	
Food secure (HFI 9)	5 (0.1)
Food insecure (HFI 10–15)	3464 (71.6)
Severely food insecure (HFI ≥ 16)	1372 (28.3)
Assets	
Cattle	2541 (52.5)
Any land	3429 (70.8)
Electricity	1515 (31.3)
Latrine	
None/field/bush	836 (17.3)
Open latrine	48 (1.0)
Pit latrine	202 (4.2)
Water sealed/slab	3752 (77.5)
Flush toilet	3 (0.1)
Household has at least 1 other child under age 5	1058 (21.8)

Abbreviations: HFI, household food insecurity; LSI, Living Standards Index; SD, standard deviation; WSB++, wheat‐soy blend ++.

Mean (SD) DDS was 3.7 (1.4), and 55% of the 24‐month‐old children met the MDD criteria (Figure 1). Almost all the children had consumed staple grains (97%), and 72% consumed fruits and vegetables in the past 24 h. More than half of the children had eaten meat, and around a third of children legumes, dairy or eggs. Around 80% of children reported eating snacks/desserts in the past 24 h.

### 3.2 Feeding Behaviours and Diet

About 70% of mothers reported high responsive feeding, and 34% of mothers reported high forceful feeding. Responsive feeding was positively associated with DDS, higher odds of meeting MDD and higher consumption of meat. Conversely, forceful feeding was negatively associated with DDS and reduced odds of meeting MDD. Forceful feeding was also associated with lower consumption of legumes, grains and vitamin A‐rich fruits/vegetables (Table 4, Figure 1).

**Table 4:** Associations between caregiver feeding behaviours and diet quality among children at 24 months of age (*n* = 4733).^a^

Diet measures	High (70.5%, *n* = 3338) versus low (29.5%, *n* = 1395) responsive feeding		High (34.0%, *n* = 1608) versus low (66.0%, *n* = 3125) forceful feeding	
	Crude	Adjusted	Crude	Adjusted
DDS, mean (SD)	3.86 (1.35)		3.76 (1.39)	
*β* (95% CI)	0.13 (0.04, 0.225)*	0.09 (0.001, 0.17)* ^,^ b	−0.07 (−0.15, 0.02)	−0.12 (−0.21, −0.04)* ^,^ c
MDD (DDS ≥ 4), %	59.8		55.8	
Odds ratio (95% CI)	1.23 (1.09, 1.40)**	1.17 (1.02, 1.33)* ^,^ d	0.90 (0.79, 1.01)	0.82 (0.72, 0.93)* ^,^ e

Abbreviations: β, standardised regression coefficient; CI, confidence interval; DDS, dietary diversity score; MDD, minimum dietary diversity; SD, standard deviation.

* *p* ≤ 0.05

** *p* ≤ 0.001.

^a^ Data were collected from 4733 mother–child dyads; those with feeding behaviour responses were included in the analysis. Feeding behaviours were dichotomised: high responsive (*n* = 3338), low responsive (*n* = 1395), high forceful (*n* = 1608) and low forceful (*n* = 3125).

^b^ Multivariable linear regression model examining the association between DDS and responsive feeding, adjusting for maternal age, maternal education, child age, child sex, paternal occupation, fever in the last 3 months, morbidity in the past 7 days, living standards index, electricity, having another child under age 5, and supplementation group, and accounting for clustering within study design.

^c^ Multivariable linear regression model examining the association between DDS and forceful feeding, adjusting for maternal education, paternal education, child age, child sex, living standards index, type of latrine, and supplementation group, and accounting for clustering within study design.

^d^ Multivariable logistic regression examining the association between MDD and responsive feeding, adjusting for maternal education, paternal education, child age, child sex, living standards index, type of latrine, and supplementation group, and accounting for clustering within study design.

^e^ Multivariable logistic regression examining the association between MDD and forceful feeding, adjusting for maternal education, child age, child sex, paternal occupation, fever in the last 3 months, morbidity in the past 7 days, living standards index, and accounting for clustering within study design.

### 3.3 Feeding Behaviours and Growth

In multivariable models, responsive feeding was not associated with any differences in anthropometric measures (*p* > 0.05) (Tables 5 and 6). However, forceful feeding was negatively associated with WAZ and WLZ. Children of mothers reporting high forceful feeding also had higher odds of being wasted or underweight. No significant relationship was seen with LAZ or stunting (Tables 5 and 6). When looking at whether HFI modifies the relationship between forceful feeding and WAZ or WLZ, we found that in the severely food insecure group, the odds of being underweight (OR: 1.75; 95% CI: 1.37–2.24) was higher than children in food insecure families (OR: 1.27; 95% CI: 1.10–1.47). Similarly, the odds of being wasted (OR: 1.81; 95% CI: 1.36–2.41) were higher in the severely food insecure group than the food insecure group (OR: 1.47; 95% CI: 1.21–1.76).

**Table 5:** Association between caregiver feeding behaviours and child anthropometry at 24 months of age (*n* = 4733).^a^

Growth measures	Mean (SD)	High (70.5%, *n* = 3338) versus low (29.5%, *n* = 1395) responsive feeding		Mean (SD)	High (34.0%, *n* = 1608) versus low (66.0%, *n* = 3125) forceful feeding	
		*β* (95% CI)			*β* (95% CI)	
		Crude	Adjusted		Crude	Adjusted
LAZ	−1.75 (0.94)	0.07 (0.01, 0.13)*	0.04 (−0.02, 0.10)^b^	−1.78 (0.97)	−0.01 (−0.06, 0.05)	−0.04 (−0.10, 0.02)^f^
WAZ	−1.79 (0.94)	0.05 (−0.01, 0.11)	0.03 (−0.03, 0.09)^h^	−1.89 (0.95)	−0.14 (−0.19, −0.08)**	−0.16 (−0.22, −0.11)** ^,^ j
WLZ	−1.21 (0.93)	0.02 (−0.04, 0.08)	0.02 (−0.05, 0.08)^c^	−1.34 (0.93)	−0.20 (−0.25, −0.14)**	−0.20 (−0.25, −0.14)** ^,^ d
Length cm/months	81.3 (3.05)	0.26 (0.06, 0.45)*	0.15 (−0.03, 0.33)^g^	81.3 (3.10)	0.11 (−0.08, 0.29)	−0.13 (−0.31, 0.06)^i^
Weight kg/months	9.67 (1.11)	0.07 (0.004, 0.14)*	0.04 (−0.03, 0.10)^k^	9.58 (1.11)	−0.11 (−0.18, −0.04)*	−0.18 (−0.24, −0.12)** ^,^ e

Abbreviations: β, standardised regression coefficient; CI, confidence interval; LAZ, length‐for‐age *z*‐score; SD, standard deviation; WAZ, weight‐for‐age *z*‐score; WLZ, weight‐for‐length *z*‐score.

* *p* ≤ 0.05

** *p* ≤ 0.001.

^a^ Data were collected from 4733 mother–child dyads; those with feeding behaviour responses were included in the analysis. Feeding behaviours were dichotomised: high responsive (*n* = 3338), low responsive (*n* = 1395), high forceful (*n* = 1608) and low forceful (*n* = 3125).

^b^ Multivariable linear regression examining the association between LAZ and responsive feeding, adjusting for maternal education, paternal education, child age, child sex, living standards index, type of latrine, and supplementation group, and accounting for clustering within study design.

^c^ Multivariable linear regression examining the association between WLZ and responsive feeding, adjusting for maternal education, paternal education, child age, child sex, living standards index, type of latrine, and supplementation group, and accounting for clustering within study design.

^d^ Multivariable linear regression examining the association between WLZ and forceful feeding, adjusting for maternal education, paternal occupation, child age, child sex, symptoms of respiratory illness in the past 3 months, fever in the past 3 months, morbidity in the past 7 days, living standards index, electricity, and supplementation group, and accounting for clustering within study design.

^e^ Multivariable linear regression examining the association between weight and forceful feeding, adjusting for maternal education, paternal occupation, child age, child sex, fever in the past 3 months, morbidity in the past 7 days, living standards index, electricity, having another child under age 5, and supplementation group, and accounting for clustering within study design.

^f^ Multivariable linear regression examining the association between LAZ and forceful feeding, adjusting for maternal education, paternal occupation, child age, child sex, morbidity in the past 7 days, living standards index, electricity, having another child under age 5, and supplementation group, and accounting for clustering within study design.

^g^ Multivariable linear regression examining the association between length and responsive feeding, adjusting for maternal education, paternal education, child age, child sex, living standards index, type of latrine, and supplementation group, and accounting for clustering within study design.

^h^ Multivariable linear regression examining the association between WAZ and responsive feeding, adjusting for maternal education, paternal education, child age, child sex, living standards index, type of latrine, and supplementation group, and accounting for clustering within study design.

^i^ Multivariable linear regression examining the association between length and forceful feeding, adjusting for maternal education, paternal occupation, child age, child sex, living standards index, electricity, having another child under age 5, and supplementation group, and accounting for clustering within study design.

^j^ Multivariable linear regression examining the association between WAZ and forceful feeding, adjusting for maternal education, paternal occupation, child age, child sex, fever in the past 3 months, morbidity in the past 7 days, living standards index, electricity, having another child under age 5, and supplementation group, and accounting for clustering within study design.

^k^ Multivariable linear regression examining the association between weight and responsive feeding, adjusting for maternal education, paternal education, child age, child sex, living standards index, type of latrine, and supplementation group, and accounting for clustering within study design.

**Table 6:** Association between caregiver feeding behaviours and prevalence of stunting, wasting and underweight among children at 24 months of age (*n* = 4733).^a^

Growth measures	%	High (70.5%, *n* = 3338) versus low (29.5%, *n* = 1395) responsive feeding		%	High (34.0%, *n* = 1608) versus low (66.0%, *n* = 3125) forceful feeding	
		Odds ratio (95% CI)			Odds ratio (95% CI)	
		Crude	Adjusted		Crude	Adjusted
Stunting (LAZ < −2)	39.1	0.91 (0.80, 1.04)	0.93 (0.83, 1.06)^d^	40.0	1.02 (0.90, 1.15)	1.06 (0.93, 1.22)^e^
Underweight (WAZ < −2)	41.4	0.96 (0.85, 1.09)	0.99 (0.87, 1.13)^f^	46.3	1.33 (1.18, 1.50)**	1.38 (1.22, 1.56)** ^,^ g
Wasting (WLZ < −2)	19.2	1.01 (0.86, 1.18)	1.02 (0.87, 1.20)^b^	24.0	1.57 (1.36, 1.82)**	1.55 (1.33, 1.81)** ^,^ c

Abbreviations: CI, confidence interval; LAZ, length‐for‐age *z*‐score; WAZ, weight‐for‐age *z*‐score; WLZ, weight‐for‐length *z*‐score.

* *p* ≤ 0.05

** *p* ≤ 0.001.

^a^ Data were collected from 4733 mother–child dyads; those with feeding behaviour responses were included in the analysis. Feeding behaviours were dichotomised: high responsive (*n* = 3338), low responsive (*n* = 1395), high forceful (*n* = 1608) and low forceful (*n* = 3125).

^b^ Multivariable logistic regression examining the association between wasting and responsive feeding, adjusting for maternal education, child age, child sex, type of latrine, and supplementation group, and accounting for clustering within study design.

^c^ Multivariable logistic regression examining the association between wasting and forceful feeding, adjusting for maternal education, child age, child sex, fever in the past 3 months, morbidity in the past 7 days, electricity, and supplementation group, and accounting for clustering within study design.

^d^ Multivariable logistic regression examining the association between stunting and responsive feeding, adjusting for maternal education, paternal education, child age, child sex, living standards index, type of latrine, and supplementation group, and accounting for clustering within study design.

^e^ Multivariable logistic regression examining the association between stunting and forceful feeding, adjusting for maternal education, paternal occupation, child age, child sex, living standards index, electricity, having another child under age 5, and supplementation group, and accounting for clustering within study design.

^f^ Multivariable logistic regression examining the association between underweight and responsive feeding, adjusting for maternal education, paternal education, child age, child sex, living standards index, type of latrine, and supplementation group, and accounting for clustering within study design.

^g^ Multivariable logistic regression examining the association between underweight and forceful feeding, adjusting for maternal education, child age, child sex, fever in the past 3 months, living standards index, electricity, having another child under age 5, and supplementation group, and accounting for clustering within study design.

## 4 Discussion

In rural northwestern Bangladesh, in a population where undernutrition is high, we found associations between caregiver responsive or forceful feeding behaviours and child dietary and anthropometric status at 24 months of age. Responsive feeding behaviours were associated with a better diet, whereas forceful feeding behaviours were associated with poorer dietary and anthropometric outcomes.

We found that use of more forceful feeding behaviours was associated with about 30% lower odds of eating vitamin‐A‐rich fruits and vegetables, 30% lower odds of eating grains, and 14% lower odds of eating lentils compared to use of less forceful feeding. Similar findings have been reported in other studies (Bhandari et al. 2004; Hotz and Gibson 2005; Penny et al. 2005; Ruel et al. 1999) with positive relations found between the exposure to a responsive feeding intervention and higher intake of key nutrients or complementary foods. For example, a responsive feeding intervention by Bhandari et al. (2004) found significantly higher energy intakes from complementary foods in their intervention group at 9 months (mean [SD]: 1556 [1109] vs. 1025 [866] kJ; *p* < 0.001] and 18 months (mean [SD]: 3807 [1527] vs. 2577 [1058] kJ; *p* < 0.001). Similarly, a cluster‐randomised trial in Peru found that fewer children in the responsive feeding intervention group failed to meet dietary intake requirements for energy, iron and zinc as compared to the controls (Penny et al. 2005).

In addition to the key findings above, our findings suggest that dietary and growth risks related to the use of nonresponsive feeding behaviours (i.e., force feeding) were stronger than that of a beneficial relation with responsive feeding. For example, children of mothers reporting forceful feeding had 38% greater odds of being underweight and 55% greater odds of wasting as compared to those reporting lower forceful feeding behaviours. They also had lower DDS and 18% reduced odds of meeting MDD. Children of mothers reporting responsive feeding had higher DDS and 17% increased odds of meeting MDD but showed no increased odds of improved nutritional status. This is consistent with previous evidence suggesting that coercive feeding behaviours (e.g., restriction, pressure to eat, ‘clean plate’ policy) are associated with poorer diet quality (Chen et al. 2021; Vaughn et al. 2017).

Qualitative work done in this population (Chowdhury et al. 2023, unpublished data) suggests that the way children are fed is driven by an overarching concern among caregivers that their child is not eating enough, and more forceful tactics, as deemed necessary, are employed to ensure intake. Given that this is cross‐sectional data, the causality is unknown, and it is possible that thinner children may be more likely to be force‐fed with maternal perception of child size driving this finding. This scenario was likely, as we found a strong association with underweight and wasting, but not stunting. Alternatively, previous studies have shown that use of nonresponsive, controlling feeding behaviours are associated with lower acceptance of food (Flax et al. 2013), which could possibly contribute to lower intake and subsequently poorer growth, and found responsive feeding behaviours to be associated with higher acceptance of food (Flax et al. 2013). Studies in Bangladesh have also found children's appetites to be associated with caregiver feeding behaviours and undernutrition (Naila et al. 2018, 2021), as malnutrition is also associated with lower appetite (Brown, Creed‐Kanashiro, and Dewey 1995).

A strength of the present study is that it includes data from a large complementary feeding trial with appropriate dietary and anthropometric measurements as well as measures of numerous confounders and effect modifiers. Another strength is that the CFBQ was adapted from existing questionnaires but validated in this population, so it is specific to this context. The 11‐item questionnaire was brief and easy to administer, increasing the ease and feasibility of assessing feeding behaviours in this setting. This paper adds on to a limited amount of work that has been done in similar low‐income populations around scale development. Aside from a few recent studies measuring responsive feeding in Indonesia, Cambodia, Sri Lanka and Bangladesh (Black et al. 2022; Pallewaththa et al. 2021; Purwaningrum et al. 2020; Sall et al. 2020), most studies measuring feeding behaviours have been conducted in higher income settings. Conversely, study limitations include brevity of the CFBQ, preventing assessment of other feeding behaviours (e.g., restriction) and fully understanding behaviours that exist with the broader construct of responsive and nonresponsive feeding. Another limitation is the cross‐sectional design which excludes dimensions of temporality and directionality of the relations examined. Additionally, self‐reported maternal pressure to eat needs to be measured carefully in Bangladesh, as it may be capturing more socially desirable practices (e.g., responsivity) than negative or coercive practices.

## 5 Conclusions

In an undernourished setting, we found 34% of mothers exhibited forceful feeding behaviours that were linked to poorer dietary and growth outcomes. Conversely, we found that 70% exhibited responsive feeding behaviours that were linked to better dietary and growth outcomes. Our findings highlight the need for integrating responsive feeding messages within the context of complementary feeding education and counseling, paired with rigorous evaluation to follow the long‐term influence of these messages on children dietary and growth patterns.

## Author Contributions

Zaynah T. Chowdhury, Kristen M. Hurley, Saijuddin Shaikh, Sucheta Mehra, Hasmot Ali, Abu Ahmed Shamim and Parul Christian designed research. Zaynah T. Chowdhury conducted research. Zaynah T. Chowdhury analysed data. Zaynah T. Chowdhury, Kristen M. Hurley and Parul Christian wrote the paper. Zaynah T. Chowdhury had primary responsibility for final content. All authors have read and approved the final manuscript.

## Conflicts of Interest

The authors declare no conflicts of interest.

## Data Availability

The data that support the findings of this study are available from the corresponding author upon reasonable request.
